# Post-extracorporeal Membrane Oxygenation (ECMO) Bilateral Urolithiasis in an Adolescent With B-cell Acute Lymphoblastic Leukemia (ALL): A Case Report

**DOI:** 10.7759/cureus.108489

**Published:** 2026-05-08

**Authors:** Sebastian G Tobia González, Gaayana A Raju, Leon I Smith-Harrison

**Affiliations:** 1 Urology, Driscoll Children's Hospital, Corpus Christi, USA

**Keywords:** acute kidney injury, acute lymphoblastic leukemia, critical illness, ecmo, extracorporeal membrane oxygenation, multidisciplinary care, pediatric urolithiasis, ureteral calculi, ureteroscopy

## Abstract

Acute kidney injury and metabolic derangements are common after extracorporeal membrane oxygenation (ECMO), yet their potential contribution to urinary stone formation is rarely described. Critically ill patients may develop lithogenic conditions related to tubular injury, oliguria, prolonged immobilization, catabolism, infection, and alterations in urinary solute handling. We report the case of a 13-year-old girl with B-cell acute lymphoblastic leukemia undergoing induction chemotherapy who developed Pseudomonas septic shock requiring prolonged extracorporeal support with venoarterial ECMO followed by venovenous ECMO. Her course was further complicated by acute respiratory distress syndrome, bilateral pneumothoraces, and prolonged intensive care unit hospitalization.

During recovery, computed tomography revealed bilateral renal and ureteral calculi with moderate left hydroureteronephrosis. Despite significant stone burden, the patient remained clinically stable without urinary tract infection, preserved renal function, and gradual radiographic improvement. Because of ongoing chemotherapy, immunosuppression, pulmonary risk, and fluctuating procedural candidacy, management required repeated multidisciplinary reassessment involving pediatric urology, oncology, pulmonology, nephrology, and the patient’s family. Initial decompression and staged ureteroscopic intervention were considered; however, serial imaging demonstrated partial spontaneous migration of calculi, allowing temporary conservative management with tamsulosin and close surveillance. Persistent left-sided impaction and limited further migration ultimately supported definitive bilateral ureteroscopy with laser lithotripsy and short-term stenting once medically optimized.

This case highlights the complex balance between surgical urgency and systemic risk in post-ECMO patients with urolithiasis. It also raises the possibility that ECMO-related renal injury and critical illness physiology may contribute to the formation of low-density or matrix-type stones. Individualized multidisciplinary decision-making is essential in medically fragile patients.

## Introduction

Urinary stone disease in children has increased in incidence over recent decades and is now recognized as an important cause of pain, obstruction, infection, and healthcare utilization. Although metabolic abnormalities remain the most common predisposing factor, stone formation in medically complex children is frequently multifactorial and may involve dehydration, recurrent infection, immobility, nutritional disturbances, medication exposure, and structural or functional urinary tract abnormalities. Children with severe systemic illness represent a particularly challenging subgroup in whom standard treatment algorithms may not be easily applicable [1-4].

Extracorporeal membrane oxygenation (ECMO) is a life-saving form of cardiopulmonary support used in refractory cardiac or respiratory failure. However, ECMO is associated with substantial renal morbidity, including fluid overload, acute kidney injury (AKI), and persistent tubular dysfunction. The mechanisms of post-ECMO renal injury are multifactorial and include ischemia-reperfusion injury, systemic inflammation, hemolysis, microvascular dysfunction, and exposure to nephrotoxic therapies. These factors may alter urinary solute handling, reduce urine flow, and create a lithogenic environment, although urinary stone formation after ECMO has been rarely discussed in the literature [5-8].

Patients recovering from prolonged critical illness may also develop additional risk factors for calculi, including immobilization-related hypercalciuria, catabolism, metabolic acidosis, concentrated urine, recurrent antibiotic exposure, and urinary stasis. In immunocompromised patients receiving chemotherapy, management becomes even more complex because the risks of infection, anesthesia, thrombocytopenia, and treatment interruption must be carefully weighed against the risks of urinary obstruction or future sepsis [9-15].

We present the case of an adolescent with B-cell acute lymphoblastic leukemia (ALL) who developed bilateral renal and ureteral calculi after prolonged ECMO support for septic shock. Her management required repeated reassessment and close coordination among pediatric urology, oncology, pulmonology, nephrology, intensive care, and family caregivers. This case highlights both the possible pathophysiologic links between ECMO-related renal injury and stone formation and the importance of individualized decision-making in medically fragile pediatric patients. 

## Case presentation

A previously healthy adolescent female patient was diagnosed with high-risk B-cell ALL and initiated systemic therapy according to a contemporary pediatric leukemia protocol. Her disease was characterized by high-risk clinical and molecular features, although end-of-induction measurable residual disease was negative. Early in treatment, her oncologic course was complicated by severe Pseudomonas septic shock with cardiopulmonary failure requiring escalation to venoarterial extracorporeal membrane oxygenation, followed by prolonged venovenous ECMO support during the same hospitalization.

Her pediatric intensive care unit course was further complicated by acute respiratory distress syndrome, necrotizing pneumonia, bilateral pneumothoraces requiring multiple chest tubes, severe malnutrition, prolonged immobilization, steroid-associated myopathy, and marked physical deconditioning. Over time, she gradually recovered with multidisciplinary rehabilitation, nutritional support, and continuation of modified leukemia-directed therapy, including immunotherapy and later maintenance treatment.

**Figure 1:**
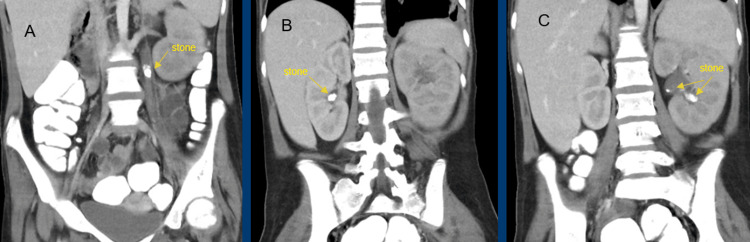
Initial CT with bilateral ureteral calculi and left hydronephrosis A) Left proximal ureteral stone. B) Right kidney stone. C) Left lower calyx stone and another one in the ureteral-pyelo junction.

During convalescence, she developed abdominal pain and emesis, prompting computed tomography (CT) of the abdomen and pelvis. Imaging demonstrated numerous calculi throughout the bilateral urinary tract, greater on the left side, with moderate hydroureteronephrosis and obstructive features (Figure 1).

Pediatric urology and nephrology were consulted. At that stage, the patient remained medically fragile, immunocompromised, and at elevated anesthetic risk because of her recent critical illness and chronic pulmonary disease. She had no active urinary tract infection or sepsis. Because the risks of urgent intervention outweighed the immediate benefits, an initial conservative strategy with close observation, serial imaging, and symptom surveillance was selected. Repeat imaging demonstrated partial migration of the ureteral calculi and improvement in obstruction without significant upstream hydronephrosis.

The case was subsequently reviewed in repeated multidisciplinary discussions involving pediatric urology, oncology, pulmonology, nephrology, the patient, and her family. Given the bilateral stone burden and the potential future risks of obstruction, infection, and renal deterioration, staged intervention was considered once her systemic condition improved. Temporary decompression with ureteral stenting followed by delayed ureteroscopy between treatment cycles was discussed as a means of minimizing procedural burden and infectious risk.

At early follow-up, the patient remained clinically stable and pain-free. Repeat imaging demonstrated partial migration of the ureteral calculi and improvement in obstruction without significant upstream hydronephrosis. Because of these favorable interval changes, planned intervention was deferred and continued conservative management was pursued. Medical expulsive and preventive strategies included tamsulosin, hydration, and urinary alkalinization/citrate supplementation.

Subsequent reassessment while she continued leukemia therapy demonstrated persistent bilateral ureteral calculi with only minimal further migration. Although she remained asymptomatic and urine cultures were negative, spontaneous passage appeared unlikely after an adequate observation period. Definitive treatment was therefore recommended once multidisciplinary clearance could be obtained and operative timing coordinated around chemotherapy requirements and pulmonary status.

She ultimately underwent cystoscopy, bilateral ureteroscopy with laser lithotripsy, and bilateral ureteral stent placement. The ureteral stents remained in place for 15 days and were subsequently removed without complication. Postoperatively, she recovered well, with no recurrent renal colic, fever, or obstructive symptoms. Follow-up renal ultrasonography demonstrated normal kidneys without hydronephrosis or definitive residual calculi, although a minimal twinkling artifact was noted.

Long-term prevention focused on maintaining hydration and citrate supplementation. Potassium citrate was prescribed, although adherence was challenging because of treatment-related nausea and poor palatability. Dietary citrate supplementation was recommended as an adjunctive strategy. During the most recent six months of follow-up, she remained free of urinary symptoms and without evidence of recurrent lithiasis. Cross-sectional imaging studies, including CT and magnetic resonance imaging obtained for surveillance of her underlying oncologic condition rather than for stone disease, likewise showed no recurrent urinary calculi or obstruction.

At the most recent oncology follow-up, she continued leukemia-directed therapy with stable blood counts, improving functional status, and coordinated outpatient care involving oncology, nephrology, pulmonology, and urology.

## Discussion

Although pediatric urolithiasis is traditionally associated with metabolic abnormalities, congenital urinary tract disorders, or recurrent infection, critically ill children may acquire multiple transient and overlapping lithogenic risk factors. Direct evidence linking ECMO itself to urinary stone formation remains limited; however, ECMO is strongly associated with AKI, renal tubular dysfunction, hemolysis, inflammation, nephrotoxic exposure, and fluid balance disturbances. These factors, together with prolonged immobilization, catabolic stress, infection, and altered urinary solute handling, may create a transiently lithogenic urinary environment. In this patient, the combination of prolonged ECMO support and severe critical illness therefore represents a biologically plausible contributor to rapid stone formation rather than a proven causal mechanism [5-8,14,15].

AKI and renal tubular dysfunction are common during and after ECMO. Reported mechanisms include ischemia-reperfusion injury, hemolysis-related pigment toxicity, inflammatory cytokine activation, endothelial dysfunction, venous congestion, and nephrotoxic exposures. Even when serum creatinine normalizes, subclinical tubular injury may persist and alter urinary handling of calcium, citrate, sodium, phosphate, and acid-base balance. Reduced urine flow during critical illness may further increase urinary supersaturation. While a direct causal relationship between ECMO and nephrolithiasis has not been firmly established, the biologic plausibility is strong and deserves further investigation [1-4,15].

Several additional factors may have contributed in this case. Prolonged immobilization can induce transient hypercalciuria through accelerated bone resorption, a recognized mechanism contributing to urinary stone formation in immobilized and critically ill patients [2,15]. Catabolic stress and metabolic acidosis may favor uric acid crystallization and hypocitraturia. Broad-spectrum antibiotic exposure and severe infection can alter the urinary milieu and may contribute to the formation of soft or matrix-type calculi. The relatively low Hounsfield unit measurements on CT in this patient raised the possibility of non-calcium stone composition, such as uric acid, drug-related, or matrix stones, although no formal stone analysis was available. This represents an important limitation, as definitive composition would have strengthened pathophysiologic interpretation and guided secondary prevention.

Management required careful balancing of urologic urgency against systemic risk. In many patients with bilateral ureteral stones and hydronephrosis, early decompression or definitive intervention would be strongly considered [9-13]. However, this patient had recent ECMO exposure, chronic pulmonary disease, immunosuppression, ongoing chemotherapy, nutritional frailty, and high anesthetic risk. Under those circumstances, immediate surgery could reasonably expose the patient to greater morbidity than short-term observation. Repeated multidisciplinary reassessment allowed management to evolve according to real-time clinical status rather than a fixed procedural algorithm.

An important teaching point from this case is that conservative management can be appropriate in selected high-risk patients, even in the presence of bilateral stone burden, when renal function is preserved, infection is absent, symptoms are controlled, and close follow-up is feasible. Serial imaging demonstrated partial migration and temporary reduction in obstruction, supporting continued observation during the highest-risk period. Once spontaneous passage became unlikely and the patient’s overall condition improved, definitive bilateral ureteroscopy with laser lithotripsy was safely performed with excellent subsequent outcomes [9-12].

This case also highlights the importance of integrating treatment schedules in children with cancer. Timing procedures between chemotherapy cycles, minimizing repeated anesthetic exposure, and coordinating perioperative infection prevention are essential considerations. Collaboration among pediatric urology, oncology, pulmonology, nephrology, anesthesia, rehabilitation services, and family caregivers was central to successful care. In medically fragile children, multidisciplinary coordination may be as important as the technical procedure itself.

The patient remained free of recurrent lithiasis and urinary symptoms during six months of follow-up, with cross-sectional imaging obtained for oncologic surveillance demonstrating no recurrent stones or obstruction. Although a longer follow-up is still necessary, this favorable course suggests that staged definitive treatment combined with preventive strategies such as hydration and citrate supplementation can be effective even after severe systemic illness.

The principal limitations of this report include its single-patient design, lack of stone composition analysis, and inability to isolate the relative contribution of ECMO versus other critical illness factors. Nonetheless, rare and complex cases such as this are valuable because they generate clinically relevant hypotheses and may help guide management in scenarios not addressed by formal guidelines. Future studies evaluating renal tubular biomarkers, urinary metabolic profiles, and stone risk after ECMO in pediatric survivors would be particularly informative.

## Conclusions

This case highlights that urinary stone disease may emerge as a clinically significant complication during recovery from prolonged critical illness and extracorporeal support in pediatric oncology patients. In children recovering from ECMO, multiple concurrent factors, including renal tubular injury, reduced urinary flow, immobilization, metabolic stress, infection, and medication exposure, may create a transiently lithogenic state even in the absence of traditional predisposing conditions.

Management should be individualized and guided by the balance between urologic urgency and overall medical risk. In selected high-risk patients who are clinically stable, carefully monitored conservative management may allow temporary deferral of intervention until systemic status improves. Once medically optimized, definitive endourological treatment can be performed safely and effectively. Successful outcomes in this setting depend on close multidisciplinary collaboration among pediatric urology, oncology, pulmonology, nephrology, anesthesia, and family caregivers. Greater awareness of potential post-ECMO urinary complications may support earlier recognition, timely referral, and improved long-term renal follow-up in survivors of critical illness.
